# ATTRv in Lazio-Italy: A High-Prevalence Region in a Non-Endemic Country

**DOI:** 10.3390/genes12060829

**Published:** 2021-05-28

**Authors:** Marco Luigetti, Valeria Guglielmino, Giovanni Antonini, Carlo Casali, Marco Ceccanti, Maria Grazia Chiappini, Laura De Giglio, Vincenzo Di Lazzaro, Antonio Di Muzio, Mariangela Goglia, Maurizio Inghilleri, Luca Leonardi, Roberto Massa, Elena Maria Pennisi, Antonio Petrucci, Emanuela Proietti, Marianna Rispoli, Mario Sabatelli, Marco Di Girolamo

**Affiliations:** 1 Fondazione Policlinico Universitario A. Gemelli IRCCS, UOC Neurologia, 00168 Rome, Italy; guglielmino.valeria@gmail.com; 2 Università Cattolica del Sacro Cuore, Sede di Roma, Largo A. Gemelli 8, 00168 Roma, Italy; mario.sabatelli@unicatt.it; 3 Unit of Neuromuscular Diseases, Department of Neurology, Mental Health and Sensory Organs (NESMOS) SAPIENZA University, Sant’Andrea Hospital, 00189 Rome, Italy; giovanni.antonini@uniroma1.it (G.A.); luca.leonardi@uniroma1.it (L.L.); 4 Department of SBMC, Sapienza University, 00161 Rome, Italy; carlo.casali@uniroma1.it; 5 Neuromuscular Rare Disease Center, Department of Human Neurosciences, University of Rome La Sapienza, 00189 Rome, Italy; marco.ceccanti@uniroma1.it (M.C.); maurizio.inghilleri@uniroma1.it (M.I.); 6 Fatebenefratelli Foundation—’San Giovanni Calibita’ Fatebenefratelli Hospital, Clinical Pathophysiology Center, 00186 Rome, Italy; mariagrazia.chiappini@fbf-isola.it (M.G.C.); emanuela.proietti@fbf-isola.it (E.P.); marco.digirolamo1946@gmail.com (M.D.G.); 7 San Filippo Neri Hospital, Neuromuscular and Rare Neurological Diseases Center, Neurology Unit, 00135 Rome, Italy; degiglio@gmail.com (L.D.G.); elenamaria.pennisi@aslroma1.it (E.M.P.); 8 Unit of Neurology, Neurobiology, Department of Medicine, University Campus Bio-Medico of Rome, 00128 Rome, Italy; v.dilazzaro@unicampus.it; 9 Centro Malattie Neuromuscolari, Clinica Neurologia, Ospedale Clinicizzato Chieti, 66100 Chieti, Italy; antoniodimuzio1@gmail.com (A.D.M.); mariannarispoli92@gmail.com (M.R.); 10 Neuromuscular Diseases Unit, Department of Systems Medicine, Tor Vergata University of Rome, 00133 Rome, Italy; mariangelagoglia@gmail.com (M.G.); massa@uniroma2.it (R.M.); 11 Center for Neuromuscular and Neurological Rare Diseases, Neurology and Neurophysiology Unit, San Camillo Forlanini Hospital, 00152 Rome, Italy; AnPetrucci@scamilloforlanini.rm.it; 12 Centro Clinico NEMO Adulti, 00168 Rome, Italy

**Keywords:** ATTRv, prevalence, amyloid

## Abstract

Hereditary transthyretin amyloidosis (ATTRv, v for variant) prevalence in Italy, a non-endemic region, has been established by ATTRv amyloidosis Italian Registry. However, values of prevalence were extremely heterogeneous, considering different regions. To properly establish the prevalence of the disease in the Lazio region, a survey was sent to university regional hospitals and to main regional hospitals, in order to collect all affected patients regularly followed. We identified 100 ATTRv patients and, considering a Lazio population of 5.8/million, we estimated a ATTRv prevalence of 17.2/million. The ATTRv amyloidosis Italian Registry reported a prevalence of 8.0/million in Lazio, while our survey showed a value of double this. Our survey documented a high-prevalence for a non-endemic country. The increased awareness of the disease among general practitioners and medical specialists is a fundamental step to reduce the diagnostic delay and start an effective treatment of this disease.

## 1. Introduction

Hereditary transthyretin amyloidosis (ATTRv, v for variant) is a severe, heterogeneous multisystem condition with prevalent peripheral nervous system impairment, due to mutations in the transthyretin (TTR) gene [1,2]. The condition, presenting as an adult-onset, autosomal-dominant disease with variable penetrance, is characterized by extracellular deposition of amyloid fibrils in different organs [1,2]. Besides the peripheral nerves, the heart, kidney, gastro-intestinal system, and eyes may also be involved, leading to a life-threatening, multisystem disease with huge clinical variability and course, and death within 10 years on average [1,2].

Depending on the geographic distribution, a wide variability in age at onset and clinical presentation of ATTRv is described [1,3]. Generally, patients from endemic areas, such as Portugal, have an early-onset (<50 years) disease with initial involvement of small nerve fibers, while in non-endemic areas, patients present with a late-onset (>50 years) progressive axonal polyneuropathy [1,3]. Recently, the prevalence in Italy, a non-endemic region, has been established by the ATTRv amyloidosis Italian Registry [4]. However prevalence varies significantly in different regions [4].

## 2. Materials and Methods

To properly establish the prevalence of ATTRv in the Lazio region a survey was sent to University regional hospitals and to several regional hospitals (Fondazione Policlinico A. Gemelli-IRCCS, Umberto I Hospital, Sant’Andrea Hospital, Fatebenefratelli Hospital, Tor Vergata Hospital, San Camillo Forlanini Hospital, San Filippo Neri Hospital, Campus Biomedico Hospital, and ICOT Hospital), including all referral Centres for ATTRv, in order to collect all affected patients who are in regular follow-up. Gender, current age, mutation, type of onset (early vs. late), presence of familial history, phenotype (neurological; cardiologic; or mixed), and geographical origin of the family were collected. We also requested the number of pre-symptomatic carriers followed in each Centre.

## 3. Results

**Figure 1:**
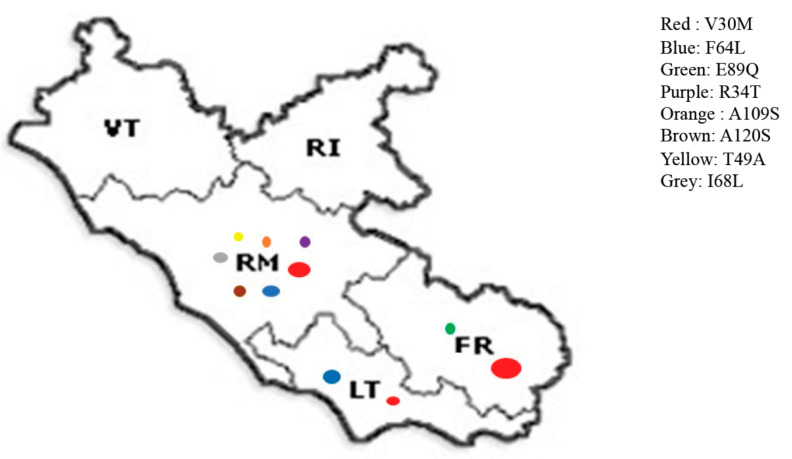
Distribution of TTR mutations in Lazio region among different provinces. Diameter of circles is proportional to number of patients.

The survey results are summarized in Table 1. All Centres replied to survey. We identified 100 ATTRv patients and, considering the Lazio population of 5.8/million, an ATTRv prevalence of 17.2/million was estimated. The most common clinical phenotype was neurological or mixed. A positive familial history was retrieved in only half of the patients. Three mutations were more frequently observed in the region (Figure 1). Half of the patients carried the V30M, and the majority of these cases came from Lazio. A quarter of the patients had the F64L mutation and all of these had families coming from southern Italy. The third most common mutation was E89Q (14%), all with ancestry from Sicily. We also identified 73 pre-symptomatic carriers.

## 4. Discussion

Prevalence of ATTRv is extremely variable around the world [4]. High prevalence was reported in endemic countries (such as Portugal or Sweden) with the highest prevalence reported in northern Portugal (1631.2/million) and northern Sweden (1040/million) [1,5]. Considering non-endemic countries, a prevalence of 7.52/million was reported in France, while a prevalence of 1.48/million was found in Germany. [5,6] In Italy, the prevalence estimated by the ATTRv amyloidosis Italian Registry is 4.33/million, with considerable differences among regions, varying from 2.5/million in Piedmont to 9.3/million in Sicily [4].

The ATTRv amyloidosis Italian Registry reported a prevalence of 8.0/million in Lazio, while our survey showed a doubled value [4]. It is likely our survey, involving more hospitals (not always included in the Registry), was able to catch almost all diagnosed patients followed in our region. However, the real number of ATTRv in our region could be also higher, considering not only overlooked diagnoses but also pre-symptomatic carriers regularly followed in each Centre.

Considering the different mutations, we found a high proportion of V30M mutation in Lazio; the great majority of the pedigree of these patients came from Lazio region, confirming the existence of an autochthonous cluster of V30M in this region. All V30M were distributed in the south of Lazio (Frosinone or Latina), and in the province of Rome. Interestingly, we did not find any ATTRv patient from the provinces of Rieti and Viterbo, in the north of Lazio region; however we cannot exclude overlooked diagnoses in this area.

We found two additional mutations (F64L and E89Q) widely distributed in Lazio. However, the pedigrees of these patients come from southern Italy, namely Sicily, for E89Q, confirming data reported by the ATTRv amyloidosis Italian Registry [4]. Migration of the southern Italian population to Rome in the recent years may explain these data.

## 5. Conclusions

ATTRv is rare and disabling disease, but today many therapies are available for this condition. Our survey confirmed the presence of a V30M cluster in Lazio, and reported a high-prevalence for a non-endemic country. The increased awareness of the disease among general practitioners and medical specialists is a fundamental step to reduce the diagnostic delay and start an effective treatment in ATTRv.

## Figures and Tables

**Table 1:** Demographic and clinical characteristics of hATTR Lazio patients.

	All Patients	V30M	F64L	E89Q	R34T	I68L	A120S	A109S	T49A
**Number of patients**	100	53 (53%)	23 (23%)	14 (14%)	3 (3%)	3 (3%)	2 (2%)	1 (1%)	1 (1%)
**M/F**	63/37 (1.7)	37/16 (2.3)	1/22 (0.04)	5/9 (0.55)	0/3 (0)	2/1 (2)	0/2 (0)	M	F
**Age (mean;**	69.42;	69.7;	71.0;	69.4;	51.7;	74.3;	70;	78	54
**median;**	70.5;	70;	72;	70.5;	58;	73;	70;		
**standard deviation;**	10.9;	10.9;	8.2;	13.4;	10.9;	9.1;	5.8;		
**range)**	39–87	47–87	51–83	43–84	39–58	73–84	66–74		
**Early onset**	17 (17%)	8 (15%)	3 (13%)	3 (21.4%)	2 (66.7%)	1 (33.3%)	0	Late onset	Late onset
**vs.**	vs.	vs.	vs.	vs.	vs.	vs.	vs.		
**Late onset**	83 (83%)	45 (85%)	20 (87%)	11 (78.6%)	1 (33.3%)	2 (66.7%)	2 (100%)		
**Family history**	58 (58%)	35 (66%)	11 (47.8%)	4 (28.6%)	2 (66.7%)	3 (100%)	2 (100%)	yes	not available
**Phenotype**	Cardiologic:	Cardiologic:	Cardiologic:	Cardiologic:	Cardiologic:	Cardiologic:	Cardiologic:	Neuropathic	Neuropathic
	7 (7%)	1 (1.9%)	0	1 (7.1%)	2 (66.7%)	3 (100%)	0		
	Neuropathic:	Neuropathic:	Neuropathic:	Neuropathic:	Neuropathic:	Neuropathic:	Neuropathic:		
	45 (45%)	28 (52.8%)	12 (52.17%)	2 (14.3%)	0	0	1 (50.0%)		
	Mixed:	Mixed:	Mixed:	Mixed:	Mixed:	Mixed:	Mixed:		
	48 (48%)	24 (45.3%)	11 (47.8%)	11 (78.6%)	1 (33.3%)	0	1 (50.0%)		
**Familial origin** **from Italy ** **(Northern ** **vs.** **Centre** **vs.** **Southern)**	Northern: 0	Northern: 0	Northern: 0	Northern: 0		Northern: 0	Northern: 0		
								
	Centre:	Centre:	Centre:	Centre:		Centre:	Centre:		
	47 (47%)	42 (79.2%)	2 (8.7%)	0		1 (33.3%)	2 (100%)		
	(Lazio: 46%)	(Lazio: 77.4%)	(Lazio: 8.7%)			(Lazio: 33.3%)	(Lazio: 100%)		
								
	Southern:	Southern:	Southern:	Southern:		Southern:	Southern:		Southern
	36 (36%)	4 (7.5%)	17 (74%)	12 (85.7%)		2 (66.7%)	0		(Sicily)
	(Sicily: 19%; Campania:	(Campania: 5.6%)	(Calabria: 21.7%;	(Sicily: 85.7%)		(Sicily: 66.7%)			
	6%; Calabria: 6%;		Apulia: 21.7%; Sicily:						
	Puglia: 5%)		17.4%;						
			Campania: 13.0%)						
	Abroad: 1 (1%)	Abroad: 1 (1.9%)							
								
	Not available:	Not available:	Not available:	Not available:	Not available			Not available	
	16 (16%)	6 (11.3%)	4 (17.39%)	2 (24.3%)					

***Legend to the Table:*** Northern Italy includes Lombardy, Piedmont, Veneto, Alto-Adige, Liguria, Emilia-Romagna, and Tuscany; central Italy includes Lazio, Abruzzo, and Molise; southern Italy includes Campania, Sicily, Apulia, and Calabria. Most frequent regions of familial origin are specified.

## Data Availability

The data presented in this study are available on request from the corresponding author.
